# Supplementary figures and images for: Chitosan Nanoparticle-Mediated Delivery of Curcumin Suppresses Tumor Growth in Breast Cancer
Source: Nanomaterials (Basel). 2024 Jul 31;14(15):1294. doi: 10.3390/nano14151294 (PMC11314098; doi:10.3390/nano14151294)

**A**

**Curcumin**

**Cur-CHNP**

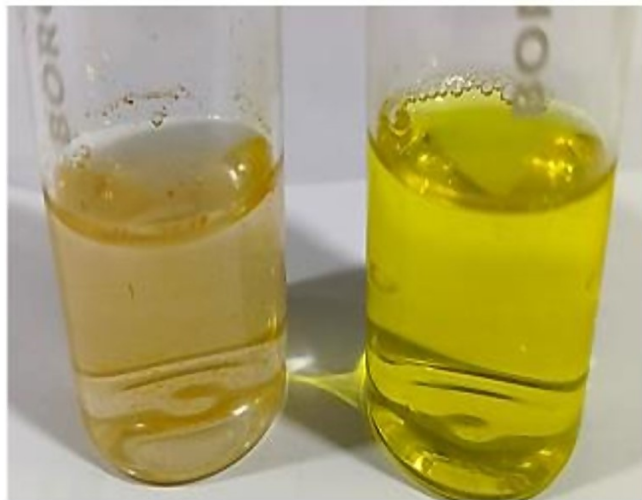

**B**

**Curcumin**

**Cur-CHNP**

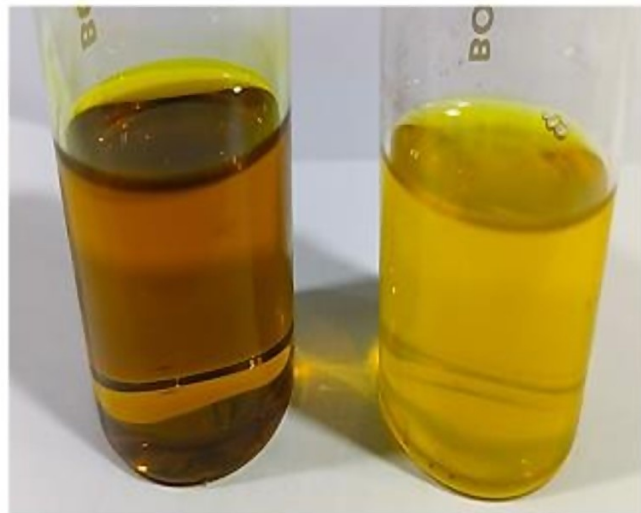

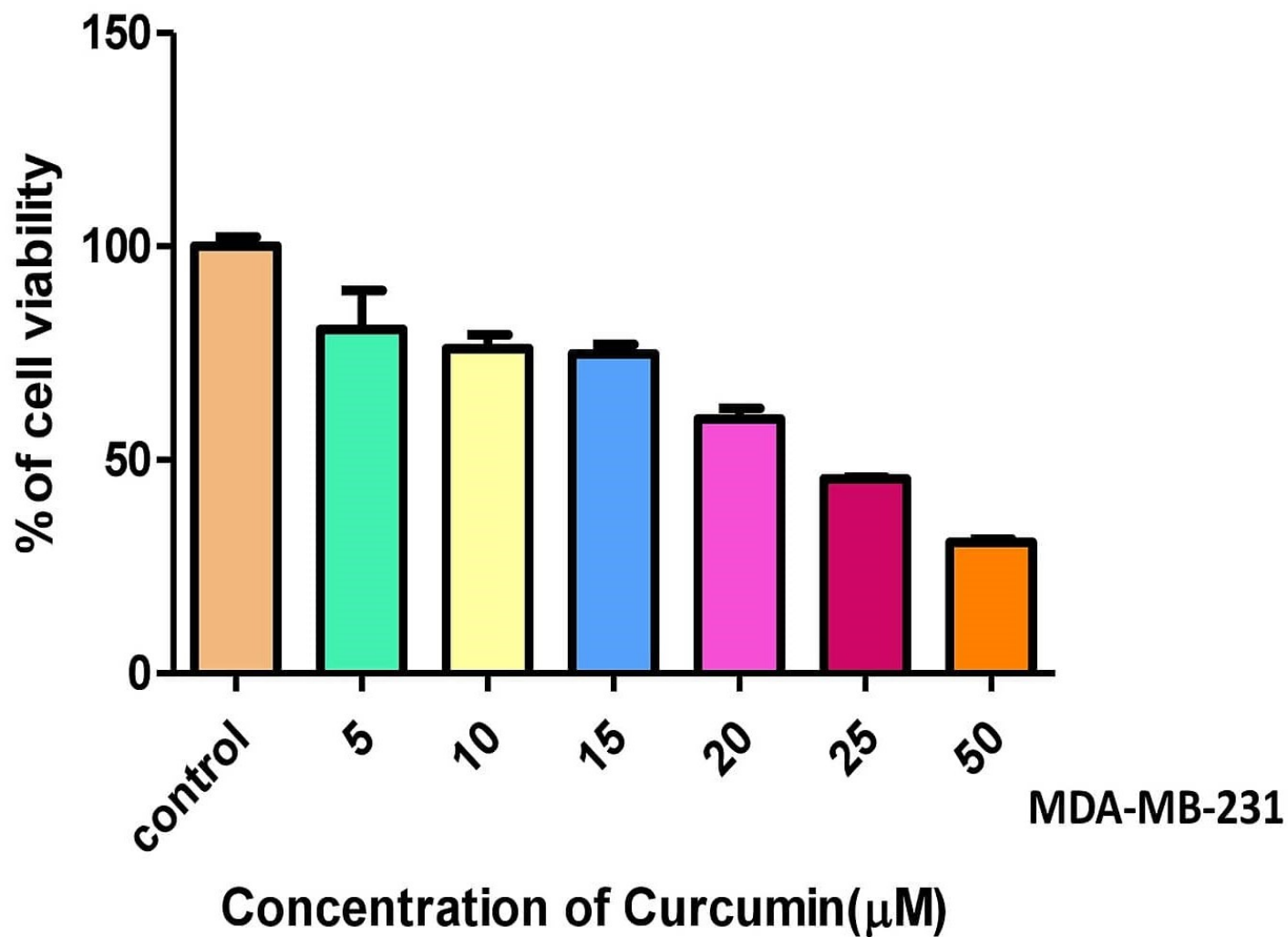

Transmittance

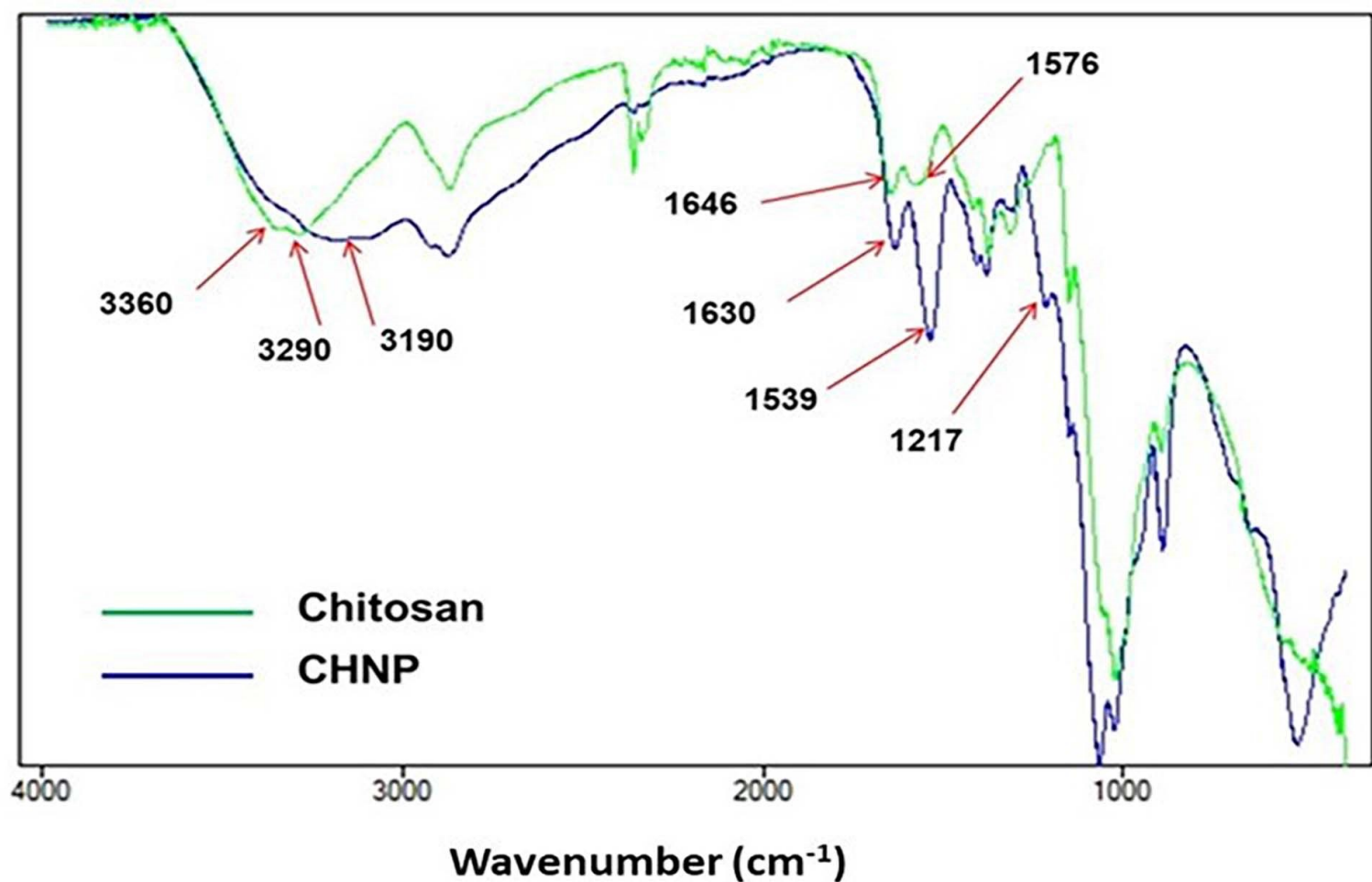

A

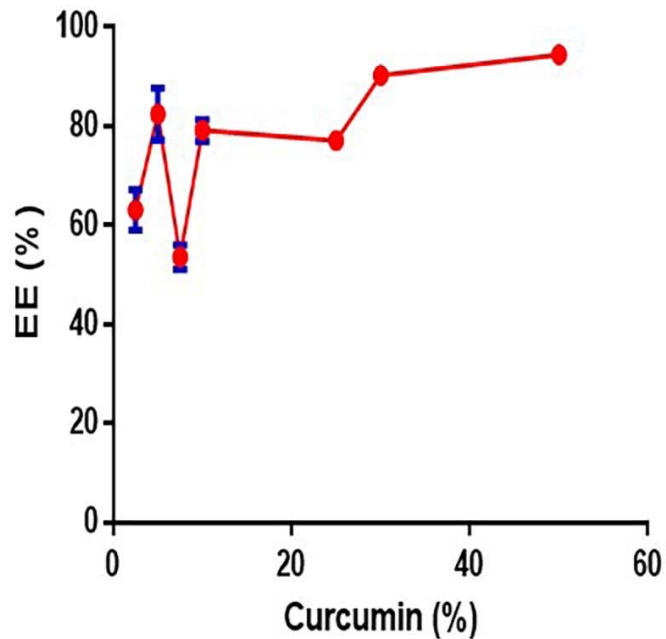

B

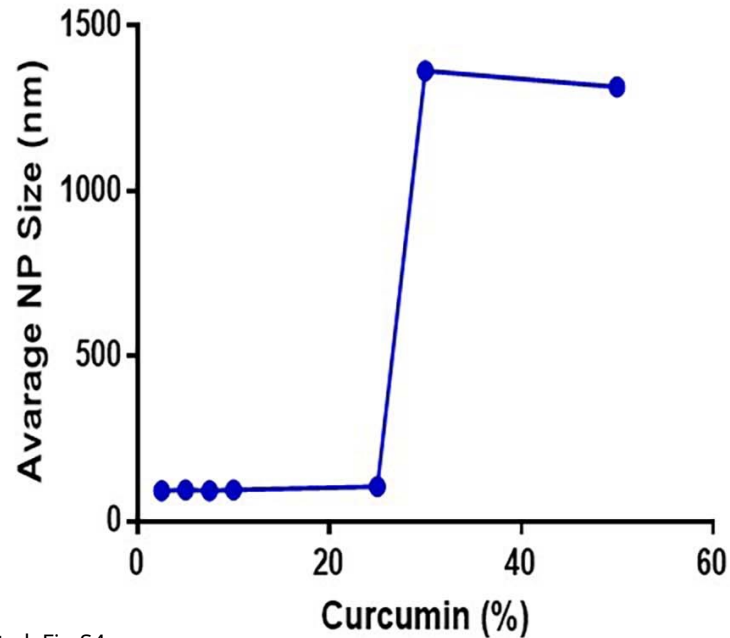

**A**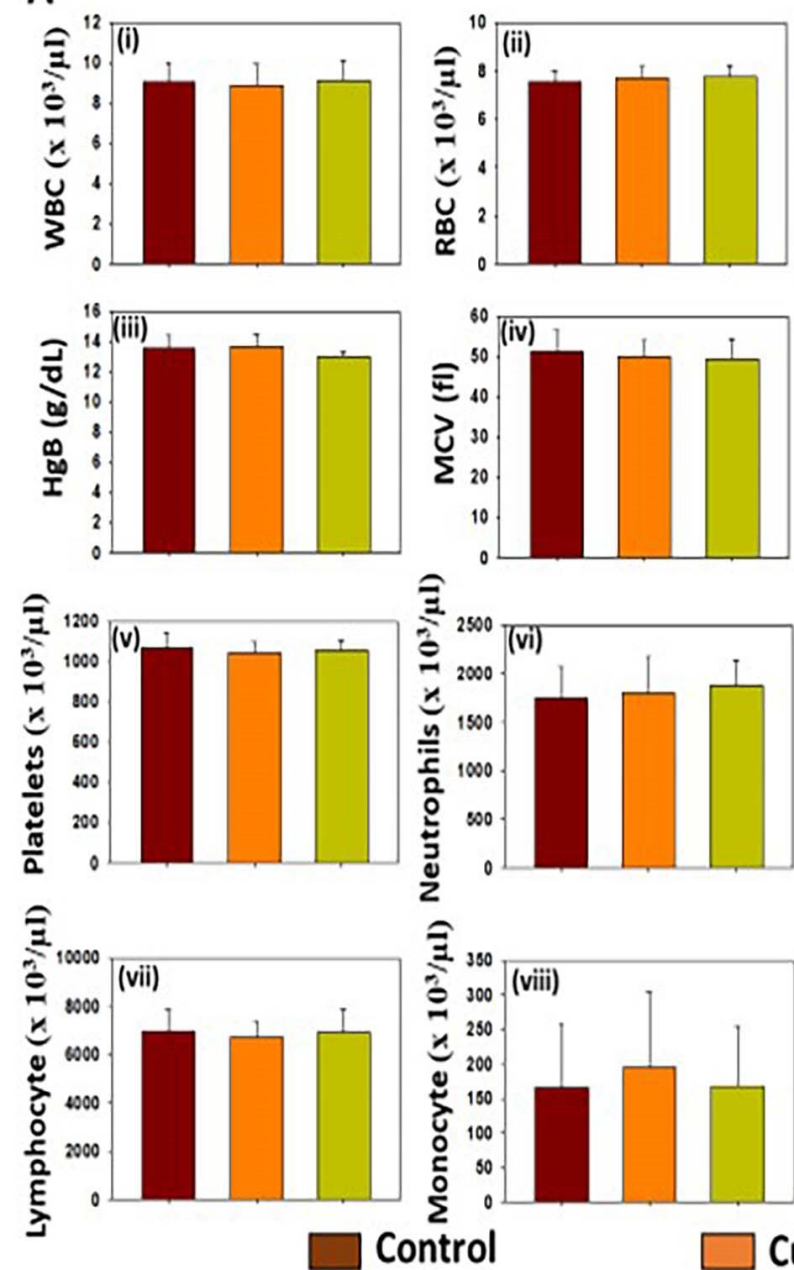**B**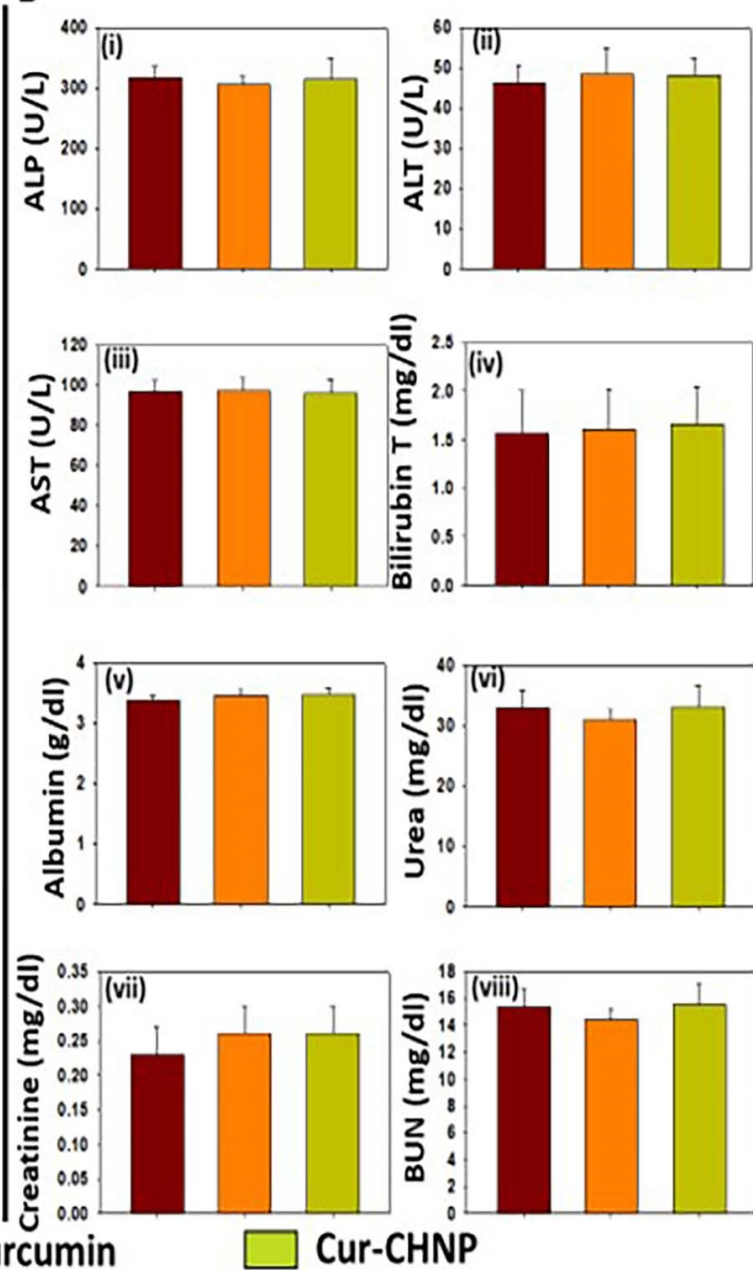

Supplement: Supplementary file 1 [file nanomaterials-14-01294-s001.zip › nanomaterials-3098737-supplementary.pdf]
